# Atypical Chemokine Receptor 1 polymorphism cannot be used as an indicator of liver fibrosis progression in Hepatitis C virus positive patients

**DOI:** 10.12669/pjms.335.12590

**Published:** 2017

**Authors:** Lin-Nan Shao, Shu-Ting Zhang, Shi-Hang Zhou, Wei-Jian Yu, Ming Liu

**Affiliations:** 1 Lin-Nan Shao, Dalian Blood Center, Dalian, Liaoning, 116001, China; 2 Shu-Ting Zhang, Dalian Blood Center, Dalian, Liaoning, 116001, China; 3 Shi-Hang Zhou, Dalian Blood Center, Dalian, Liaoning, 116001, China; 4 Wei-Jian Yu, Dalian Blood Center, Dalian, Liaoning, 116001, China; 5 Ming Liu, Department of Cell Biology, Dalian Medical University, Dalian, 116044, China

**Keywords:** Atypical chemokine receptor 1, Hepatitis C virus, Liver damage, Liver fibrosis, Polymorphism, Serum liver related markers

## Abstract

**Background & Objective:**

Atypical chemokine receptor 1(ACKR1) represents an atypical chemokine receptor that can bind promiscuously to various chemokines. Chemokines play a crucial role to recruit leukocyte subsets migration through the endothelium and into liver against the virus during the progression of hepatitis C virus (HCV) infection. Most HCV positive patients can lead to liver fibrosis. Hyaluronic acid (HA), laminin (LN), collagen IV(C-IV) and amino-terminal pro-peptide of Type-III pro-collagen (PIII NP) are indices of the extent of liver fibrosis. The aim of this study was to investigate the association between ACKR1 polymorphism and liver fibrosis with these four serum liver markers in HCV positive patients.

**Methods:**

From April 2015 to December 2015, a total of 210 patients (109 males and 101 females) with chronic HCV infection at Dalian Infectious Hospital were recruited to participate in this study. ACKR1 genotyping was using TaqMan probes method. HA, LN, C-IV and PIII NP were detected by using diagnostic kits.

**Results:**

We compared serum levels of HA, LN, C-IV and PIII NP between FY^*^A/FY^*^A and FY^*^A/FY^*^B patients and the differences were not significant (*P*=0.905, *P*=0.298, *P*=0.880 and *P*=0.470, respectively).

**Conclusions:**

This study has attempted to elucidate the role of ACKR1 polymorphism in liver fibrosis progression of HCV infection, our results demonstrated that ACKR1 polymorphism is not directly associated with the fibrogenesis in HCV positive patients.

## INTRODUCTION

Hepatitis C virus (HCV) is a global public health problem, which affects 130 to 150 million individuals. With time, most subjects with chronic HCV infection develop hepatic fibrosis, and may progress cirrhosis and end stage liver disease. Liver fibrosis is resulted from the loss of normal liver cell function due to excess deposition of various components that constitute the extracellular matrix (ECM) involving molecular and histological rearrangement of various types of collagens, proteoglycans, structural glycoproteins and hyaluronic acid (HA).^1^ Liver biopsy has always been regarded as the “gold standard” for the diagnosis of liver fibrosis and evaluation of its severity. However, liver biopsy has many short comings.^2^ In recent years, some studies have proposed non-invasive, serum-based biomarkers: HA, laminin (LN), collagen IV (C-IV) and amino-terminal pro-peptide of Type-III pro-collagen (PIIINP) as indices of the extent of liver fibrosis in chronic liver diseases.^3^

The Duffy blood group antigens, also called Duffy antigen receptor for chemokines (DARC), recently renamed atypical chemokine receptor 1 (ACKR1). Two major alleles of ACKR1 for Asians were described: FY^*^A and FY^*^B.^4^ Furthermore, two main functions have been postulated for ACKR1. One is as a sink or scavenger receptor for chemokines and the other is involved in the transcytosis of chemokines across endothelial cells and presents them to leukocytes.^5^ Chemokines, a large family of leukocyte chemoattractants, have become increasingly recognized as important mediators of hepatic inflammation and injury.^6^ ACKR1 polymorphisms have attracted substantial interest in recent years because of their critical roles in diseases, such as breast cancer, *Plasmodium vivax* malaria and HIV.^7^-^9^ However, we would like to emphasize that only little is known about the correlation between polymorphism of ACKR1 and hepatic fibrosis processes with HCV infection. In order to enrich the knowledge of this field, we aimed to investigate the association between ACKR1 polymorphism and liver fibrosis progression with HA, LN, C-IV and PIIINP.

## METHODS

From April 2015 to December 2015, a total of 210 patients (109 males and 101 females) with chronic HCV infection at Dalian infectious hospital were recruited to participate in this study. To avoid sampling error-introducing bias, patients with HCV infection satisfied the following criteria: presence of HCV antibody and HCV RNA with abnormal liver function tests; free of ascites; Hepatitis B virus or alcohol related liver diseases could be ruled out for them; no significant heart, brain, lung, and kidney complications. The fasting venous blood samples were collected. Informed consent was obtained from all individual participants included in the study. All procedures were performed in accordance with ethical standards of the responsible committee on human experimentation (institutional and national) and with the Helsinki Declaration. This research did not receive any specific grant from funding agencies in the public, commercial, or not for profit sectors.

### ACKR1 genotyping

Genomic DNA was extracted by a commercially available DNA isolation kit on MagCore^®^ Automated Nucleic Acid Extractor (RBCBioscience, Taipei, Taiwan). The ACKR1 was genotyped by 5’-nuclease assay (NA) with TaqMan-minor groove binding (MGB) probes method. The primers and probes were synthesized by Applied Biosystems (Table-I). The PCR mixtures included 1μL of purified genomic DNA, 10μL of 2×TaqMan Universal PCR Master Mix II (Applied Biosystems, Foster City, CA, USA), 0.9 μL of each primer (20 μM), 0.2 μL of each probes (20 μM) and 6.8 μL of distilled water in a final reaction volume of 20 μL. The 5’-NA was performed on the ABI Prism 7300 sequence detection system (Applied Biosystems) using a cycle of 95°C for 10 min, followed by 40 cycles at 95°C for 15 s and at 60°C for 1 min. The results were sorted into three distinct groups, corresponding to the three genotypes, homozygous FY^*^A/FY^*^A, FY^*^B/FY^*^B and heterozygous FY^*^A/FY^*^B.

**Table-I:** The sequences of TaqMan-minor groove binding probes and primers.

*Primers and probes*	*Sequence (5'-3')*	*Tm^†^ (°C)*
Probe for FY*A	FAM-AGACTATGGTGCCAAC-MGB	65
Probe for FY*B	VIC-AGACTATGATGCCAACC-MGB	66
Forward primer	TGTGAATGATTCCTTCCCAGATG	59.8
Reverse primer	CACTGGTGAGGATGAAGAAGGG	59.6

*Note:* The polymorphic sites are underlined.

† Melting temperature.

### Serum biochemical measurement

The diagnostic kits for the HA, LN, C-IV and PIII NP were provided by the Autobio Diagnostics Co., Ltd. (Zhengzhou, Henan Province, China). The operations were performed according to the manufacturer’s instructions.

### Statistical Analysis

Genotype and allele frequencies were calculated by direct counting method. The classical test for Hardy-Weinberg equilibrium (HWE) was examined. The Kolmogorov-Smirnov test was used to check the data of these markers for Gaussian distribution. If Gaussian distribution, the serum markers between patients with different ACKR1 genotypes were analyzed by t-test; otherwise, were analyzed by Mann-Whitney *U*-test. The *P*-value of less than 0.05 (*P*<0.05) was considered statistically significant difference. The statistical analyses performed using SPSS version 21.0.

## RESULTS

**Fig.1:**
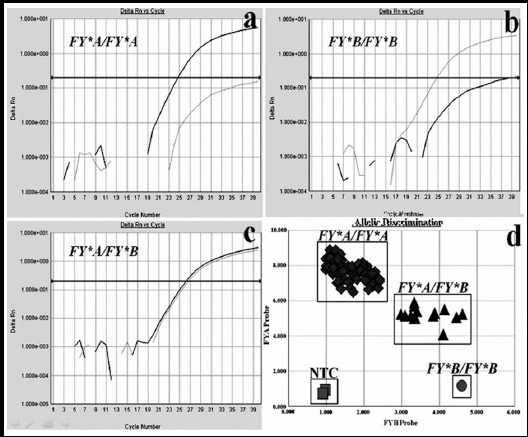
Representative amplification curves of homozygous FY*A/FY*A (a), homozygous FY*B/FY*B (b) and heterozygous FY*A/FY*B (c). End-point fluorescent signals from several samples (d): Homozygous FY*A/FY*A showed an increased fluorescence along the Y-axis, homozygous FY*B/FY*B along the X-axis, whereas heterozygous FY*A/FY*B showed an increase in fluorescence intensity along both the X-axis and Y-axis. Black curves: fluorescent intensity of dye FAM. Gray curves: fluorescent intensity of dye VIC. NTC: No Template Control.

**Fig. 2:**
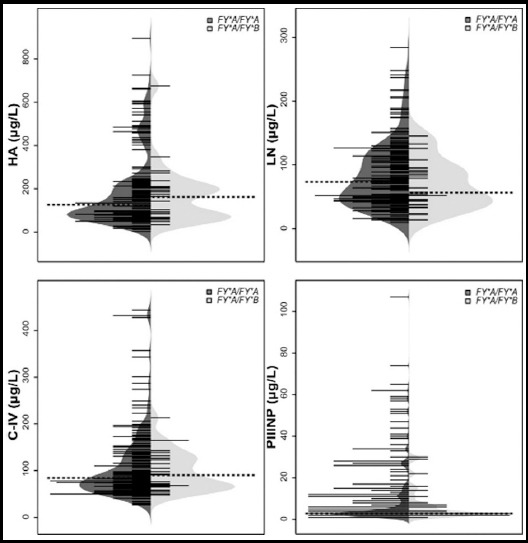
Beanplot of the distributions of hyaluronic acid (HA), laminin (LN), collagen IV (C-IV) and amino-terminal pro-peptide of Type-III pro-collagen (PIIINP) in 210 HCV positive patients between FY*A/FY*A and FY*A/FY*B genotypes. Dashed lines denote the medians.

Overall, 210 patients with HCV infection were genotyped by using the TaqMan-MGB probes. Representative analysis results were displayed in Fig.1. The results of ACKR1 polymorphisms were shown in Table-II. No significant deviation was found from HWE (*P*=0.32). FY^*^A/FY^*^A was most predominant, FY^*^A/FY^*^B was less common, and FY^*^B/FY^*^B was not found. A beanplot is suitable for density trace and visualizing distributions of individual observations.^10^ Therefore, we used beanplots to compare the distributions of four serum fibrosis markers between FY^*^A/FY^*^A and FY^*^A/FY^*^B patients (Fig.2). The results pointed out that serum levels of HA, LN, C-IV and PIIINP did not significantly correlate between FY^*^A/FY^*^A and FY^*^A/FY^*^B patients (*P*=0.905, *P*=0.298, *P*=0.880 and *P*=0.470, respectively).

**Table-II:** ACKR1 polymorphism of HCV infected subjects.

*All subjects*	FY*A/FY*A	FY*A/FY*B	FY*B/FY*B	FY*A	FY*B
n	183	27	0	393	27
%	87.1	12.9	0	93.6	6.4

## DISCUSSION

More than 70% of the patients who are chronically infected with HCV will develop liver fibrosis, liver cirrhosis, hepatocellular cancer and liver failure. Studies have clearly documented that early stages of fibrosis are reversible either by removal of the specific stimulus or by treatment with antifibrotic medications, whereas late stages, progressing to cirrhosis, are less reversible.^11^ Serum liver fibrosis markers which diagnose the presence of liver fibrosis and classify its severity have been paid much attention to by various scholars.^3^ Thus, analyzing the risk factors of liver fibrosis and damage progression with serum markers is valuable for not only screening high-risk patients, but also for preventing and treating the patients in the early stage. The polymorphism of ACKR1 was genotyped within 210 patients with chronic HCV infection. The genotype distribution results were consistent with the characters of Asians that FY^*^A was the most predominant allele and FY^*^B was not prevalent.^4^

Chemokines that play a crucial role to recruit leukocyte subsets migration through the endothelium and into liver to against the virus during the progression of HCV infection can be secreted directly by the liver.^12^ ACKR1 represents an atypical chemokine receptor that can bind promiscuously to various chemokines belonging to the CC and CXC families.^13^ Recent study found that ACKR1 polymorphism is associated with serum concentration of CCL2 belonging to the CC family.^14^ Moreover, the functional relevance of CCL2 has been demonstrated in experimental liver fibrosis models and patients with HCV infection.^15^ Thus, we conjectured that ACKR1 polymorphism might influence the liver fibrosis. Nevertheless, data presented herein refute this hypothesis and demonstrate thatACKR1 polymorphism cannot be used as an indicator of liver fibrosis progression in Asians with chronic HCV infection. The possible explanation for these negative associations is that liver fibrosis progression is a complex process which is affected by many other factors, and ACKR1 is responsible for only 20% of the variation in CCL2 serum levels, which might also restrict its biologic significance in hepatitis C.^14^

## CONCLUSION

This study has attempted to elucidate the role of ACKR1 polymorphism in liver fibrosis progression of HCV infection, but our results demonstrated that ACKR1 polymorphism is not directly associated with the fibrogenesis in HCV positive patients by using non-invasive serum liver related markers. This study was based on a relatively small size of subjects. Determinations of the exact functional consequences of ACKR1 polymorphism need more detailed and careful in vitro as well as in vivo studies.

### Authors` Contribution

**LN S & ST Z:** Did statistical analysis & manuscript writing.

**SH Z & WJ Y:** Did data collection & review.

**ML:** Conceived designed and final approval of manuscript.
